# A noble extended stochastic logistic model for cell proliferation with density-dependent parameters

**DOI:** 10.1038/s41598-022-12719-y

**Published:** 2022-05-30

**Authors:** Trina Roy, Sinchan Ghosh, Bapi Saha, Sabyasachi Bhattacharya

**Affiliations:** 1 Agricultural and Ecological Research Unit, Indian Statistical Institute, Kolkata, 700108 India; 2 Department of Mathematics, Government College of Engineering and Textile Technology, Berhampore, 742101 India

**Keywords:** Cellular noise, Applied mathematics, Cell division, Population dynamics

## Abstract

Cell proliferation often experiences a density-dependent intrinsic proliferation rate (IPR) and negative feedback from growth-inhibiting molecules in culture media. The lack of flexible models with explanatory parameters fails to capture such a proliferation mechanism. We propose an extended logistic growth law with the density-dependent IPR and additional negative feedback. The extended parameters of the proposed model can be interpreted as density-dependent cell-cell cooperation and negative feedback on cell proliferation. Moreover, we incorporate further density regulation for flexibility in the model through environmental resistance on cells. The proposed growth law has similarities with the strong Allee model and harvesting phenomenon. We also develop the stochastic analog of the deterministic model by representing possible heterogeneity in growth-inhibiting molecules and environmental perturbation of the culture setup as correlated multiplicative and additive noises. The model provides a conditional maximum sustainable stable cell density (MSSCD) and a new fitness measure for proliferative cells. The proposed model shows superiority to the logistic law after fitting to real cell culture datasets. We illustrate both conditional MSSCD and the new cell fitness for a range of parameters. The cell density distributions reveal the chance of overproliferation, underproliferation, or decay for different parameter sets under the deterministic and stochastic setups.

## Introduction

Cells can recolonize an empty area within a culture plate after being induced by cell-cell interaction for proliferation^1,2^. Such interaction-induced proliferation depends on cell density^3–5^. A positive interaction between two cells depends upon cellular cooperation to avail the resources such as space and food; it facilitates proliferation^6^. On the other hand, a negative interaction between a cell and growth-inhibitory molecules (e.g. mitomycin C) reduces cell population in the culture plate as an additional negative feedback apart from existing contact inhibition^7,8^. Like ecological populations, cells can experience density regulation through contact-inhibition due to crowding under a limited resource^9^. Since the cells react to even slightest fluctuations in the environment, there is a high chance to observe noise in cell proliferation for such a case^10,11^. Thus predicting the interaction-induced cell proliferation dynamics through mathematical modeling is challenging and can open new avenues in the field of the growth curve.

The widely used logistic growth model has a series of demerits in predicting this intercellular-interaction-induced proliferation^12,13^. The logistic model neither incorporates a density-dependent intrinsic growth rate (IGR) nor has an additional negative feedback term to separate the effect of growth-inhibiting molecules from the contact inhibition and limiting resource^12,14,15^. A few cooperation models have a density-dependent IGR, but the negative feedback term is absent in those growth laws^16^. On the other hand, the well-established harvesting model describes the additional negative feedback term but lacks a parametric representation of cooperation, or density-dependent IGR to describe such cell proliferation^17^. $\theta$–logistic model and some of its variations best describe only density regulation due to crowding for any population^18–20^. Therefore, combining concepts of these three modeling frameworks can synthesize a new growth curve for interactive cell-proliferation dynamics. Note that Roy et al.^15^ already incorporated linear density-dependent IGR and additional negative feedback in an extended logistic model to capture such cell proliferation. However, that extended model still lacks flexibility in terms of regulation in the density-dependence of IGR and contact inhibition under a limited resource. Most importantly, this model ignores the uncertainty present in cell proliferation resulting in an unrealistic scenario in cell dynamics.

Experimental scientists can predict and optimize the outcome of cell cultures using such a model based on evident properties of cell proliferation. Such a model can predict the stable cell density at the maximum sustainable condition and maximum cell-population fitness. Proposal of this evidence based generalized cell proliferation model also demands a comparative analysis with its preceeding models^12,21,22^ to understand its ability for portraying data properties. Exploration of a growth models’ property under a stochastic setup extends its advantage in the experimental field. It enriches the knowledge of uncertainty, the behavior of equations, and the generalized growth models^23^.

The uncertainty in cell proliferation may come from different sources and mechanisms^11^. For example, non-uniform distribution or heterogeneity in growth-inhibiting molecules can cause randomness in proliferation in a multiplicative manner, while the fluctuation in micro-environmental components can be an additive noise^24–28^. Also, the association between this heterogeneous and environmental fluctuations may cause a correlation between the multiplicative and additive noises^11,29^. Therefore, a generalized model for intercellular-interaction induced cell proliferation must have correlated multiplicative and additive noises. Since the noises and their correlation influence the growth rates, the distribution of steady states is an exciting aspect of the generalized growth law under the stochastic setup. This aspect is necessary to predict the chance of overproliferation, underproliferation, or decay of cells with intercellular-interaction-induced proliferation.

Predicting the chance of different proliferation trends requires to compare the models’ behavior under stochastic and deterministic setups, identifying the distribution pattern of sustainable cell density. For example, sustainable cell density may steadily rise if the resources are abundant^30^, but the distribution can show another pattern under random and limited resource^31^. Since the noise strength can reflect the randomness in a stochastic model, checking the similarity in these distribution patterns for strengths and correlation of noises is essential from mathematical and application-oriented perspectives. The existing models with stochastic setup mainly focus on tumor dynamics with either multiplicative or additive noise. Also, most of these models are multidimensional with assigned compartments to represent specific cell types in a given environmental setup. The specific field-based conceptualization of these models limits their prospective to be applied in a vast field as a generic stochastic growth model. The consideration of correlation between noises is also insubstantial in the literature so far.

Based on the lacunae in present growth-curves for the application in intercellular-interaction-induced cell proliferation in culture, we propose a generalized model under both deterministic and stochastic setups. We set three primary objectives for this study for further understanding the proposed models’ property: (a) To determine the maximum sustainable stable cell density under stochastic and deterministic setups; (b) To determine the cell size for attaining maximum fitness; and (c) To predict trends in proliferation and fitness of cells using a real dataset.

## Materials and methods

We start with the deterministic setup for the modeling framework and convert the model to a stochastic setup using correlated multiplicative and additive noises. We use the power series approximation around the carrying capacity on the deterministic model to find the conditional MSSCD. We also verify whether the cell population actually wants to stabilize at this conditional MSSCD value through the deterministic potential function. Again, using the same approximation technique, we find the cell density with maximum fitness. We use scratch assay data from Jin et al.^1^ to evaluate our proposed model. We fit the model using the grid-search method for evaluation based on the least residual sum of square (RSS). We use “R” and “MATLAB” softwares for simulation-based study and figures generation. For analysis of stochastic setup, we use the Fokker-Planck equation and find the cell density with maximum steady state probability density function (SSPDF). We check the distribution of conditional MSSCD for different noise intensities and correlation using SSPDF. Finally, we predict the nature of cell density distribution at steady state for long run through simulation using the determined parameter values from the model fitting.

### Deterministic model formulation

- Step I: We start to form our model from the basic logistic law, i.e., $\frac{dx(t)}{dt}=rx(t)\left( 1-\big (\frac{x(t)}{K}\big )\right)$. Here *r* is the constant IGR; *x*(*t*) is the density of the population at the time point *t* and *K* is the carrying capacity. We shall refer IGR as intrinsic proliferation rate (IPR) for cell proliferation from now on as the cell populations’ growth considered here is the proliferation only. Due to the inter-cellular interaction (especially cooperation), *r* must be density-dependent. So we consider, $r \propto x(t) ^{\alpha }$, i.e., *r* is an allometric function ($r=r_{p}x(t)^{\alpha }$) of the cell density to capture a generic density-dependent form. The parameter $\alpha$ is the regulator of inter-cellular interactions/cell-cell cooperation and $r_{p}$ is the new constant IPR. Thus, the logistic model transforms to $\frac{dx(t)}{dt}=r_{p}x(t)^{(\alpha +1)}\left( 1-\big (\frac{x(t)}{K}\big )\right)$. This equation can be treated as a generalized version of Von-Bertalanffy model^12,32^.
- Step II: Richards^20^ and Accinelli^33^ already noted that the $\frac{x(t)}{K}$ of the logistic law represents the environmental resistance and crowding effect on cell proliferation. We incorporate the concept of environmental resistance and crowding regulation of cells at the culture flask by considering $\beta$ as the allometric power of $\frac{x(t)}{K}$. Thus, the model now transforms into-
  1 $$\begin{aligned} \frac{dx(t)}{dt}=r_{p}x(t)^{(\alpha +1)}\left( 1-\big (\frac{x(t)}{K}\big )^{\beta }\right) . \end{aligned}$$
- Step III: As cells interact with growth-inhibiting molecules alongside other cells, an additional negative feedback apart from contact inhibition acts on the proliferation. This negative effect may either be a constant or depend upon the cell density. If a cell receives *c* amount of negative feedback upon interacting with growth-inhibiting molecules, the total suppression of the cell population becomes *cx*(*t*). We consider the *c* as a further allometric density-dependent negative feedback rate per cell for more generalization. Assuming $c=nx(t)^{\delta }$, the total suppression becomes $nx(t)^{(\delta +1)}$. The $\delta$ represents the regulation rate of interaction between cell and growth-inhibiting molecules to produce negative feedback. Therefore, the final deterministic model for the intercellular-interaction-induced cell proliferation dynamics is-
  2 $$\begin{aligned} \frac{dx(t)}{dt}=r_{p}x(t)^{(\alpha +1)}\left( 1-\big (\frac{x(t)}{K}\big )^{\beta }\right) - nx(t)^{(\delta +1)}. \end{aligned}$$

### Stochastic model with multiplicative and additive noises

Determining true cell density is impossible due to the stochasticity involved through the heterogeneity in various gases and temperatures in the culture flask. The randomness involved in the true data of cell density can be well captured through additive noise. The multiplicative noise is introduced to the cell proliferation due to the heterogeneous distribution of growth-inhibiting molecules, such as streptomycin and penicillin, in the culture media. We transform the proposed deterministic model (2) to a stochastic one to understand the proliferation trends under a random environment in this sub-section. Noise is one of the well-established ways to introduce stochasticity in the cell proliferation model^34–39^. However, none of the models with noise in the literature consider inter-cellular cooperation, environmental resistance, and additional negative feedback. Our proposed final deterministic model (Eq. 2) can capture all these phenomena. Also, unlike most existing models, the multiplicative noise is associated with the additional negative feedback instead of the proliferation rate in interaction-induced cell proliferation. This association is due to the heterogeneity in the growth-inhibiting molecules in cell culture. Yang et al.^40^ and d’Onofrio^41^ are the first to consider the multiplicative noise in the negative feedback rate. We also consider a similar multiplicative noise in the negative feedback rate based on the information on growth-inhibiting molecules. For the multiplicative noise, we transform *n* into $n+\varepsilon (t)$. So the Eq. (2) becomes $\frac{dx(t)}{dt}=r_{p}x(t)^{(\alpha +1)}\left( 1-\big (\frac{x(t)}{K}\big )^{\beta }\right) - nx(t)^{(\delta +1)}-x(t)^{(\delta +1)} \varepsilon (t)$. Additional noises may affect the proliferation due to the fluctuations in environmental conditions. So we consider the additive noise $\Gamma (t)$ in the cell proliferation upon interaction at time point *t*. With both additive and multiplicative noises in the proposed deterministic model, we introduce the following stochastic model-

3 $$\begin{aligned} \frac{dx(t)}{dt}=r_{p}x(t)^{(\alpha +1)}\left( 1-\big (\frac{x(t)}{K}\big )^{\beta }\right) - nx(t)^{(\delta +1)}-x(t)^{(\delta +1)} \varepsilon (t)+ \Gamma (t). \end{aligned}$$

Equation (3) is a Stratonovich stochastic differential equation. Here, $\varepsilon (t)$ and $\Gamma (t)$ are Gaussian white noises with the following properties:

$$\begin{aligned} \langle \varepsilon (t) \rangle= & {} \langle \Gamma (t) \rangle = 0, \nonumber \\ \langle \varepsilon (t) \varepsilon (t^{\prime }) \rangle= & {} 2D \delta (t-t^{\prime }),\nonumber \\ \langle \Gamma (t) \Gamma (t^{\prime }) \rangle= & {} 2Q \delta (t-t^{\prime }),\nonumber \\ \langle \varepsilon (t) \Gamma (t^{\prime }) \rangle= & {} \langle \Gamma (t) \varepsilon (t^{\prime }) \rangle = 2 \lambda \sqrt{DQ} \delta (t-t^{\prime }). \end{aligned}$$

Here, *D* and *Q* are the multiplicative noise and additive noise strengths, respectively. In other words, *D* increases if the heterogeneity in growth-inhibiting molecules increases. Similarly, *Q* can increase for a more fluctuating environment, leading to $\Gamma (t)$ increment. Here, the additive and the multiplicative noises are delta correlated. The association between the distribution of growth-inhibiting molecules and the environment of cells supports this correlation. We denote $\lambda$ as the degree of the correlation strength between these two Gaussian white noises.

#### *Remark 1*

An empirical researcher must be aware of the uncertainty resulting from a low-cost experiment. The multiplicative noise strength of the model provides an idea about the deviation from expected cell density under heterogeneity. The additive noise in the model provides an idea about the deviation from expected cell density under environmental randomness.

While dealing with cell dynamics data, one may assume the independence between the noises observed at two different time points. The errors involved in operating the instruments for cell culture may or may not be independent. Note that we must assume an independent error structure for Gaussian white noise. If the same experimenter handles the instrument, the system involves colored noise. Let us understand the colored noise definition mathematically from the perspective of cell proliferation in the Remark 2.

#### *Remark 2*

Let, $\in (t)$ and $\Gamma (t)$ are the Gaussian color noises with zero mean and the following properties:

4 $$\begin{aligned} \langle \varepsilon (t) \varepsilon (t^{\prime }) \rangle= & {} \frac{D}{\tau _{1}} exp[-\frac{|t-t^{\prime }|}{\tau _{1}}],\nonumber \\ \langle \Gamma (t) \Gamma (t^{\prime }) \rangle= & {} \frac{Q}{\tau _{2}} exp[-\frac{|t-t^{\prime }|}{\tau _{2}}],\nonumber \\ \langle \varepsilon (t) \Gamma (t^{\prime }) \rangle= & {} \langle \Gamma (t) \varepsilon (t^{\prime }) \rangle = \frac{\lambda \sqrt{DQ}}{\tau _{3}}exp[-\frac{|t-t^{\prime }|}{\tau _{3}}]. \end{aligned}$$

Here, the noises $\in (t)$ and $\Gamma (t)$ are exponentially correlated. $\tau _{1}$ is the self correlation time of the multiplicative noise and $\tau _{2}$ is the self correlation time of the additive noise. Again, $\tau _{3}$ is the cross correlation time between the additive and the multiplicative noises. When the correlation times converges to zero then the colored noise become the white noise. If we consider the color noise in our proposed model (2) then the proposed model becomes the same model mentioned in (3) but with the above mentioned properties of $\in (t)$ and $\Gamma (t)$ as represented in Eq. (4). Among the two types of noises (white and colored), we consider the white noise for cell proliferation for mathematical simplicity. Note that the assumption of independence is not always unrealistic in case of dealing the data of cell proliferation.

#### *Remark 3*

We can fit the model to the dataset only if stable equilibrium points are present. The equilibrium points and the stability analysis of the model are in the result section. In the following subsection, we describe the dataset we use for fitting and the parameter estimation procedure through the fitting.

### Dataset and fitting methodology

Jin et al.^1^ used PC-3 cell line in RPMI-1640 medium with $10\%$ fetal calf serum, at 37 °C, in $5\%$ $\text{CO}_{2}$, $95\%$ air for scratch assay. The data from the assays have three-time series for three different initial seeding densities: 12,000, 16,000, and 20,000 cells per well. The components of the culture media and its air, flask size, and temperature are the environment of PC-3 cell, which determines the *K* of our model. The 100 U/mL penicillin and 100 μg/mL streptomycin in the culture medium inhibits the unwanted fungal and bacterial growths. However, recent studies suggest penicillin can hamper the growth of cancer cells by inducing autophagy^42^. Although Streptomycin is not reported to affect mammalian cell growth directly, it has been suspected to alter metabolism of cells in-vitro^43^. So we consider these two as unintended growth-inhibiting factors determining the additional negative feedback. The initial cell population in the experiment proliferated overnight and started to interact after forming a uniform “scratch” by a wound-maker. The experimenters washed the culture twice with the fresh medium and further added 100 μL medium. Placing the plates in the incuCyte live cell imaging system, they collected the data with 2 h interval for first 18 h and then 6 h interval up to 48 h. So there are three experimental datasets for three initial seeding conditions. Each seeding condition has three replicates. The mean of the replicates for each seeding condition therefore makes up a dataset from three experimental setups.

Since the data shares the basic conceptions of the proposed model, viz., density-dependent IPR and additional negative feedback, we use these datasets to fit our model for evaluation. One way to fit the model into the dataset is through standard nonlinear regression. However, the experimental setups generated 15 data points for each seeding condition, and our model has six parameters. Therefore, the parameter estimation may not be significant through the nonlinear least-square method. Another well established technique for model fitting is grid-search technique^16,19,44,45^. We perform the entire grid search procedure using the following steps-

1. We compute relative proliferation rates (RPR) from the data sets based on the relative growth rate (RGR) estimates by Fisher^46^.
2. Choosing the grid value range for each parameter and dividing it into *h* equal partitions, we run the grid-search algorithm in “R”. There are $(h + 1)$ points in the partitioned space of each of the six parameters, $(h + 1)^{6}$, where a hexad of $r_{p}$, *K*, *n*, $\alpha$, $\beta$, and $\delta$ parameter combinations are available.
3. Computing the RSS values at each combination, we choose the hexad corresponding to the minimum RSS as the best model fit to the dataset.

Finding the parameters estimate with desired accuracy level is possible by repeated tuning the choice of grid values. The choice of parameter grids for the dataset is in Table 1. Besides the density-RPR profile, We fit and compare both logistic models as suggested by Jin et al.^1^ and our newly proposed model through this procedure for time series also.

**Table 1:** Grid values for parameter estimation through grid-search.

Seeding 1	
$r_{p}$	(0.03, 0.13, 0.23, 0.33, 0.43, 0.53, 0.63, 0.73, 0.83, 0.93)
*K*	(1.03, 1.23, 1.43, 1.63, 1.83, 2.03, 2.23, 2.43, 2.63, 2.83)
*n*	(0.0035, 0.0095, 0.0155, 0.0215, 0.0275, 0.0335, 0.0395, 0.0455, 0.0515, 0.0575)
$\alpha$	(0.90, 1.15, 1.40, 1.65, 1.90, 2.15, 2.40, 2.65, 2.90, 3.15)
$\beta$	(0.19, 0.59, 0.99, 1.39, 1.79, 2.19, 2.59, 2.99, 3.39, 3.79)
$\delta$	(0.2, 1.0, 1.8, 2.6, 3.4, 4.2, 5.0, 5.8, 6.6, 7.4)

Seeding 2	
$r_{p}$	(0.01, 0.07, 0.13, 0.19, 0.25, 0.31, 0.37, 0.43, 0.49, 0.55)
*K*	(2.22, 2.61, 3.00, 3.39, 3.78, 4.17, 4.56, 4.95, 5.34, 5.73)
*n*	(0.004, 0.014, 0.024, 0.034, 0.044, 0.054, 0.064, 0.074, 0.084, 0.094)
$\alpha$	(0.22, 0.42, 0.62, 0.82, 1.02, 1.22, 1.42, 1.62, 1.82, 2.02)
$\beta$	(0.1, 0.5, 0.9, 1.3, 1.7, 2.1, 2.5, 2.9, 3.3, 3.7)
$\delta$	(0.08, 0.32, 0.56, 0.80, 1.04, 1.28, 1.52, 1.76, 2.00, 2.24)

Seeding 3	
$r_{p}$	(0.00, 0.13, 0.26, 0.39, 0.52, 0.65, 0.78, 0.91, 1.04, 1.17)
*K*	(1.00, 2.56, 4.12, 5.68, 7.24, 8.80, 10.36, 11.92, 13.48, 15.04)
*n*	(0.0057, 0.0157, 0.0257, 0.0357, 0.0457, 0.0557, 0.0657, 0.0757, 0.0857, 0.0957)
$\alpha$	(0.30, 0.99, 1.68, 2.37, 3.06, 3.75, 4.44, 5.13, 5.82, 6.51)
$\beta$	(1.44, 2.38, 3.32, 4.26, 5.20, 6.14, 7.08, 8.02, 8.96, 9.90)
$\delta$	(0.025, 0.030, 0.035, 0.040, 0.045, 0.050, 0.055, 0.060, 0.065, 0.070)

## Results

### Stability analysis of the deterministic model

**Figure 1:**
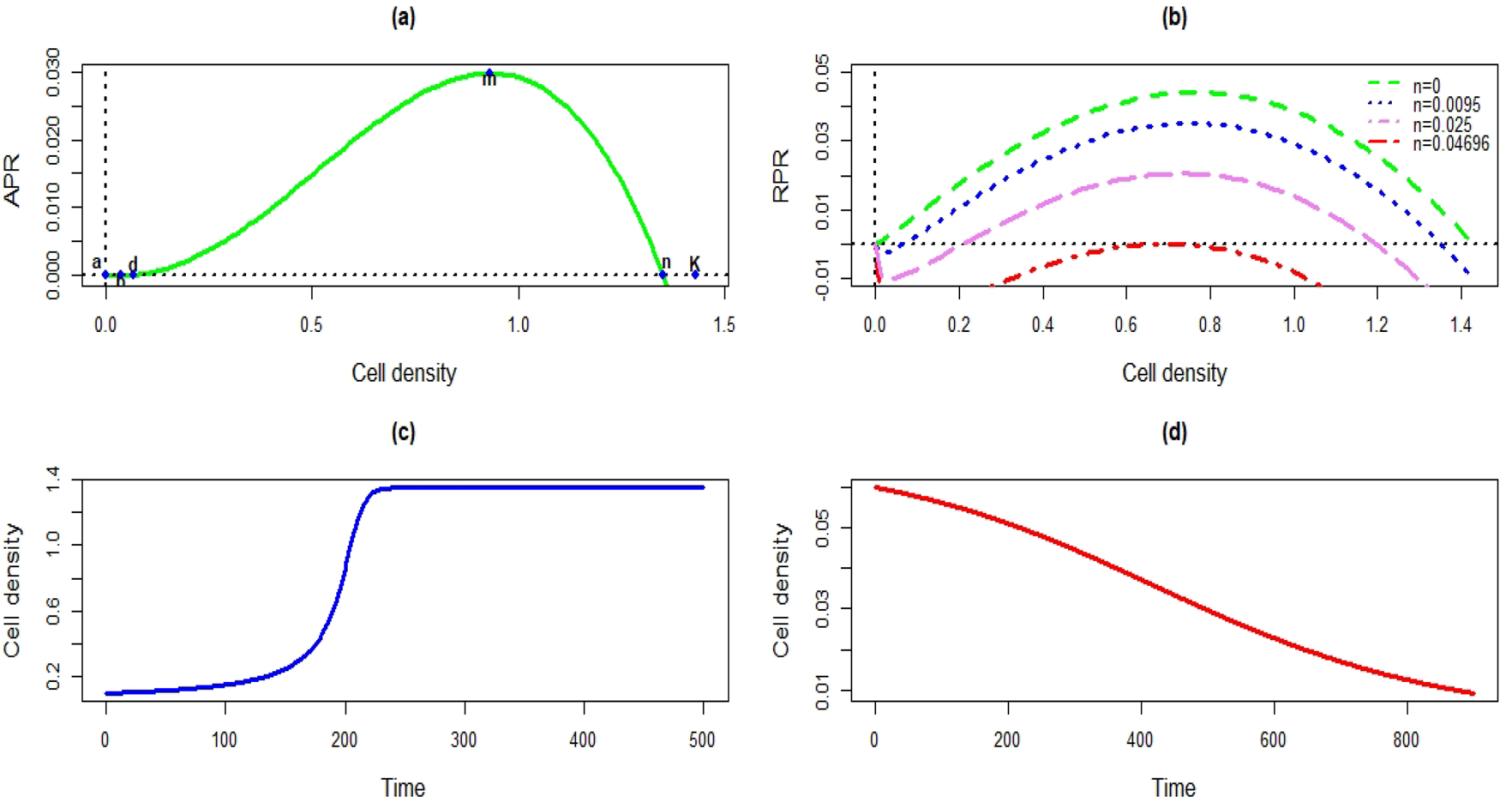
Growth dynamics of the proposed model: (**a**) Absolute proliferation rate (APR) profile considering $r_{p}=0.13$, $K=1.43$, $n=0.0095$, $\alpha =1.15$, $\beta =0.99$ and $\delta =0.2$; (**b**) RPR profiles for different *n* and other same constant model parameters; (**c**) Cell population survive for $r_{p}=0.13$, $K=1.43$, $n=0.0095$, $\alpha =1.15$, $\beta =0.99$ and $\delta =0.2$ with the initial cell density 0.1; (**d**) The population goes to extinction for the initial cell density 0.06 with the same constant parameters.

Solving $\left( x(t) \times \left( r_{p}x(t)^{(\alpha )}\left( 1-\big (\frac{x(t)}{K}\big )^{\beta }\right) - nx(t)^{(\delta )} \right) \right) =0$, we obtain two stable and one unstable equilibrium points for the model. One stable equilibrium is trivial, i.e., $x(t)=0$, another stable equilibrium point being the non-zero satisfying $\left( r_{p}x(t)^{(\alpha )}\left( 1-\big (\frac{x(t)}{K}\big )^{\beta }\right) - nx(t)^{(\delta )} \right) =0$. Figure 1a shows three different equilibrium points of the model. In addition to the equilibrium, the model has two inflection points (Fig. 1a). At these inflection points the absolute growth rates are minimum and maximum. The density vs relative proliferation rate (RPR) profile of the model shows that the model can attain negative RPR for a positive cell density, suggesting that the model can portray the Allee phenomenon (Fig. 1b). Figure 1c,d portray the proliferation and decay phases, respectively through the model.

The solution of the deterministic model finally provides two theorems.

#### **Theorem 1**

$x^{*}\approx K -K\left( \frac{\Big (\beta r_{p}K^{\alpha }+n \delta K^{\delta }\Big )-\sqrt{\Big (\beta r_{p}K^{\alpha }+n \delta K^{\delta }\Big )^{2}-2 \left( 2 \alpha \beta r_{p}K^{\alpha } +\beta (\beta -1)r_{p}K^{\alpha }+\delta (\delta -1)nK^{\delta } \right) nK^{\delta }}}{\left( 2 \alpha \beta r_{p}K^{\alpha } +\beta (\beta -1)r_{p}K^{\alpha }+\delta (\delta -1)nK^{\delta } \right) }\right)$ *is the conditional MSSCD for the intercellular-interaction-induced proliferative cells. The conditional threshold density for cell-proliferation upon interaction is* $x^{*}=K -K\left( \frac{\Big (\beta r_{p}K^{\alpha }+n \delta K^{\delta }\Big )+\sqrt{\Big (\beta r_{p}K^{\alpha }+n \delta K^{\delta }\Big )^{2}-2 \left( 2 \alpha \beta r_{p}K^{\alpha } +\beta (\beta -1)r_{p}K^{\alpha }+\delta (\delta -1)nK^{\delta } \right) nK^{\delta }}}{\left( 2 \alpha \beta r_{p}K^{\alpha } +\beta (\beta -1)r_{p}K^{\alpha }+\delta (\delta -1)nK^{\delta } \right) }\right)$ (*proof is in the* supplementary information*)*.

Allee and cooperation models are the only extended logistic law other than our model to provide a threshold population size for growth or proliferation. Our proposed model is superior to the Allee and cooperation model as it can detect the conditional threshold cell density for proliferation and regulate the density by its different parameters. For example, One may reduce the conditional threshold density by either regulating the interaction between growth-inhibiting molecules and cells ($\delta$) or reducing the inhibiting molecule concentration (*n*).

The conditional MSSCD from Theorem 1 is lower than the carrying capacity of the conventional logistic model due to growth-inhibiting molecules; it provides the expected cell density during culture in a given environment. Theorem 1 also states the set of parameters to control the cell proliferation and get the desired density during such cell cultures. A further question arises knowing this set of parameters: which one of the parameters in the expression is crucial in terms of application purpose? Since the $r_{p}$ is the constant proliferation rate for a given cell line, controlling the conditional MSSCD is not possible through $r_{p}$. We simulate the distribution of conditional MSSCD for other parametric planes to answer this question. For this, we use the parameter values obtained from the data.

#### **Theorem 2**

*The RPR is maximum at the cell density* $x^{*}= K-K\left( \frac{r_{p}\beta K^{\alpha -1}+n\delta K^{\delta -1}}{2r_{p}\alpha \beta K^{\alpha -1}+r_{p}\beta (\beta -1)K^{\alpha -1}+n\delta (\delta -1)K^{\delta -1}}\right)$ *for the concave downward profile under the condition* $r_{p}\alpha (\alpha -1){x^{*}}{}^{(\alpha -2)}-\frac{r_{p}}{K^{\beta }}(\alpha +\beta )(\alpha +\beta -1){x^{*}}{}^{(\alpha +\beta -2)}-n\delta (\delta -1){x^{*}}{}^{(\delta -2)}<0$ *(proof is in the* supplementary information*)*.

The cell density at the maximum RPR measures the fitness of the cell population and its proliferativeness. This cell density provides the idea about the extension of the proliferation phase, which is vital to predict the duration of culture and the number of observations to be taken during data collection.

#### *Remark 4*

The parameter $\alpha$ is the regulator of intercellular interactions/cell-cell cooperation. The parameter $\delta$ represents the regulator of interaction between cells and growth-inhibiting molecules to produce negative feedback. The equality of these two physical parameters indicates that the amount of signal a cell receives for proliferation is exactly the same as the signal of growth inhibition. In such a case, the environmental resistance and crowding regulator $\beta$ plays a key role in controlling the cell proliferation dynamics. The concept of conditional threshold cell density is not at all meaningful as the intercellular interactions/cell-cell cooperation regulator already reaches the value of growth inhibition regulation when $\alpha =\delta$. This concept can be well understood through the usual stability analysis as described below. It is worthy of mentioning that mathematically we have only two equilibrium points, viz., one stable and another unstable, when $\alpha =\delta$. For $\alpha =\delta$ the proposed deterministic model (2) becomes $\frac{dx(t)}{dt}=r_{p}x(t)^{(\alpha +1)}\left( 1-\big (\frac{x(t)}{K}\big )^{\beta }\right) - nx(t)^{(\alpha +1)}$. This model has two equilibrium points with zero as the unstable equilibrium point and $K(1-\frac{n}{r_{p}})^\frac{1}{\beta }$ as the stable equilibrium point under the condition $r_{p}>n$ (see the supplementary information). The cell population sustain with any positive initial cell density *x*(*t*) and try to stabilize at $x(t)= K(1-\frac{n}{r_{p}})^\frac{1}{\beta }$. Therefore, bimodality vanishes and unimodality is observed for the case $\alpha =\delta$ $r_{p}>n$. The RPR profile will be concave downward always with the maximum RPR value is at the inflection point $x(t)= K(\frac{(r_{p}-n)\alpha }{r_{p}(\alpha +\beta )})^\frac{1}{\beta }$. The deterministic potential function in this case is $U(x)=-\Big [(r_{p}-n)\frac{x^{(\alpha +2)}}{(\alpha +2)}-\frac{r_{p}}{K^{\beta }}\frac{x^{(\alpha +\beta +2)}}{(\alpha +\beta +2)} \Big ]$. The minima of this effective potential function will be at $x(t)= K(1-\frac{n}{r_{p}})^\frac{1}{\beta }$ which is the maximum stable cell density for $r_{p}>n$.

### Parameter estimation

**Figure 2:**
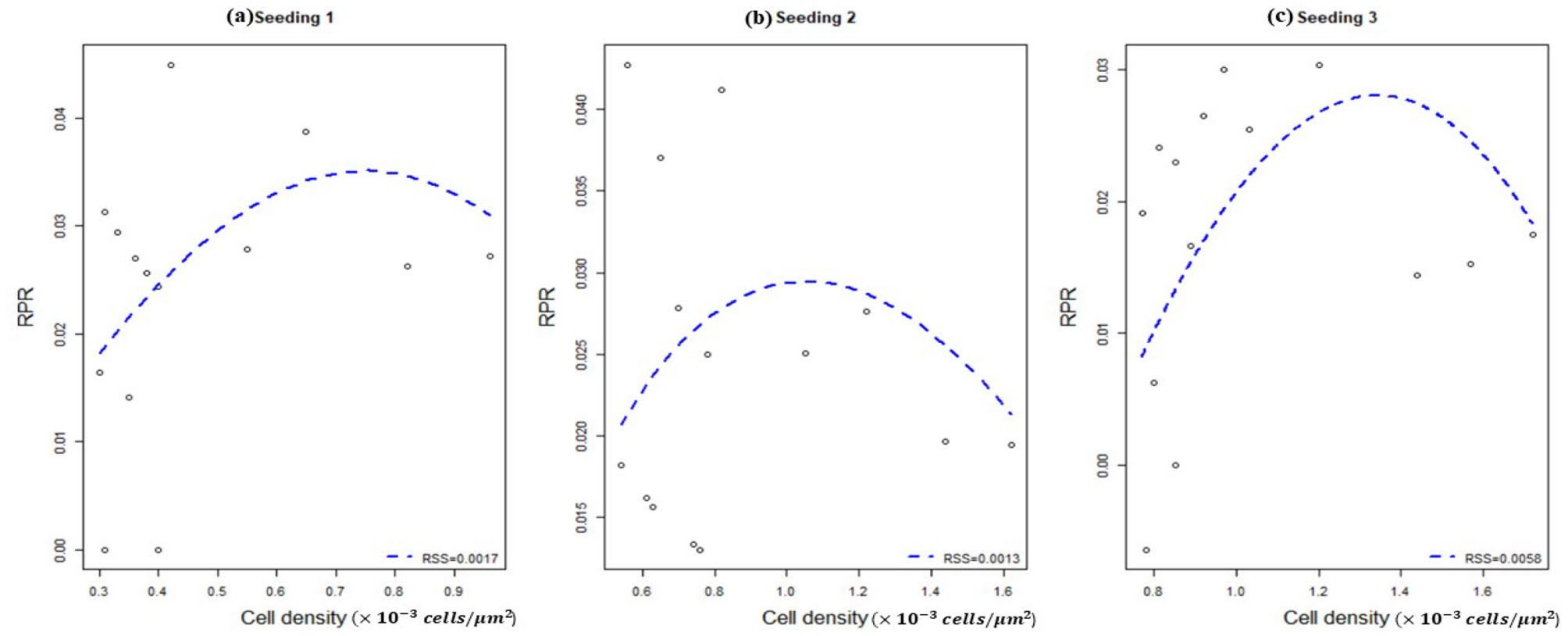
Our proposed model best fitted the cell density-RPR datasets for all of the seeding conditions generated through the grid-search method.

The density-RPR and time-density fitting to the scratch assay datasets show a lower RSS for our model than the logistic one for each of the three seeding conditions. The estimated parameters from the RPR fitting through the grid-search are in Table 2. Although the RSS for the RPR fitting of the seeding 2 is very low, the data itself is too scattered in both the upper and lower range for the small cell density. Therefore, there is a chance that regardless of the low RSS value, the fitting for seeding 2 may not reflect the actual estimates of the parameters with the bias in the data set (Fig. 2b). Nevertheless, the density-RPR fittings to the other two seeding density datasets do not suffer from bias (Fig. 2a,c).

**Table 2:** Estimated model parameters from density-RPR fitting of our model.

Seeding condition	$\hat{r_{p}}$	${\hat{K}}$	${\hat{n}}$	${\hat{\alpha }}$	${\hat{\beta }}$	${\hat{\delta }}$	RSS value in RPR profile	RSS value in density profile
1	0.13	1.43	0.0095	1.15	0.99	0.2	0.0017	0.0027
2	0.13	3.39	0.074	0.42	1.3	0.08	0.0013	0.0068
3	0.13	2.56	0.0757	0.99	1.44	0.07	0.0058	0.0185

Jin et al.^1^ suggested that their two phase logistic model may share similarities with the Allee effect. However, they did not fit the Allee model stating seeding 2 and 3 were large enough seeding densities. We calculated the conditional threshold density, conditional MSSCD, density at the minimum and maximum RPR for the model from our estimated parameters (Table 3). The conditional threshold cell density calculated from our estimated parameters confirms that the smallest initial seeding density of the dataset was greater than the conditional threshold cell density.

**Table 3:** Calculated cell densities from estimated parameters from our model fitting.

Seeding condition	Conditional threshold cell density	Conditional maximum sustainable stable cell density	Density at lowest inflection point	Density at heighest inflection point
1	0.0671	1.3507	0.0359	0.9325
2	0.2062	2.1799	0.0897	1.4152
3	0.6549	1.9761	0.3109	1.478

**Figure 3:**
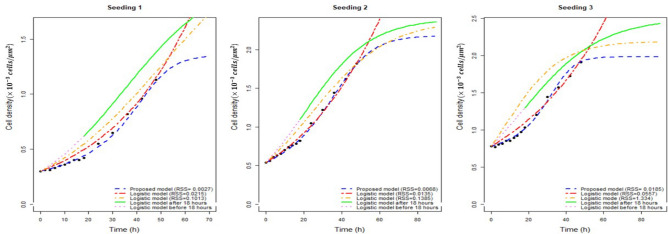
Time series solution of the proposed model and logistic law with comparative RSS for all three seeding conditions.

Figure 3 compares the portrayal of the data through our model with the fitting by Jin et al.^1^. The blue dashed line is the time-series fitting of the proposed model, and the red-colored line is the time-series fitting of the logistic model to the scratch assay data sets in the Fig. 3. The carrying capacity values are unexpectedly very high in the logistic fit, keeping the model near the exponential phase for the entire dataset. Thus the overall and two phase logistic fits are unrealistic compared to the highest cell density observed in the assay. Also, logistic fitting of the RPR profiles to the data after 18 h does not capture the whole scenario. The green solid and the violet dashed line represent the logistic time-density fit after and before 18 h density profiles respectively. The orange-colored lines in the Fig. 3 are the expected population density as per estimated parameters from the RPR fitting after 18 h data sets. Table 4 enlists all parameters for a comparison between logistic and our model fitting.

**Table 4:** Logistic model fitting with the Jin et al.^1^ estimates used in Fig. 3 with the specific colors.

Seeding 1		
Color	${\hat{r}}$	${\hat{K}}$
Red	0.028	$2.8\times 10^{10}$
Orange	0.04	2.5
Green + violet	0.051	2.1

Seeding 2		
Color	${\hat{r}}$	${\hat{K}}$
Red	0.029	8.7
Orange	0.05	2.3809
Green + violet	0.059	2.4

Seeding 3		
Color	${\hat{r}}$	${\hat{K}}$
Red	0.019	$1.6 \times 10^{10}$
Orange	0.07	2.1875
Green + violet	0.048	2.5

### Trends in cell densities under deterministic set up

**Figure 4:**
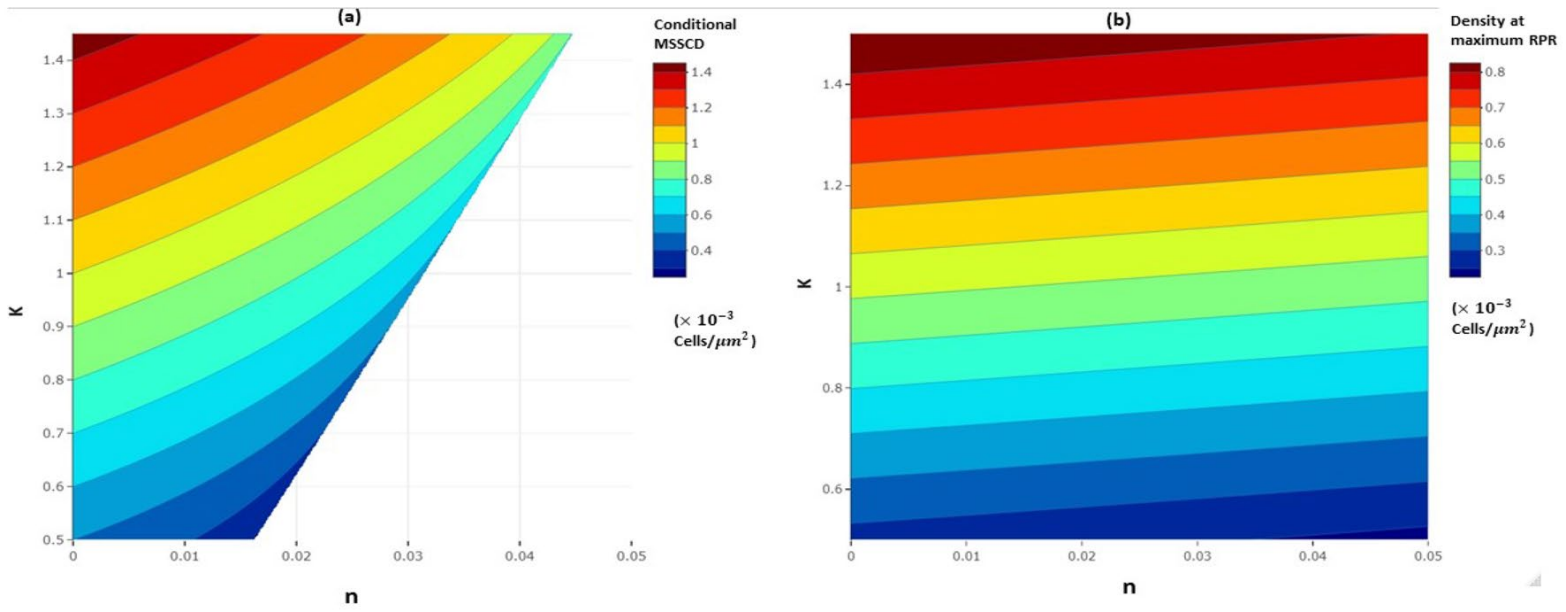
The distribution of conditional MSSCD and cell density at maximum RPR in n-K parametric plane.

The $r_{p}$ is fixed for a cell line among all the determining parameters of the conditional MSSCD. *n* and *K* vary together with the culture media, flask, and environmental setup. On the other hand, the $\alpha$, $\beta$, and $\delta$ vary together with intercellular-interactions and cellular-interaction with growth-inhibitory molecules, which depend on the medium’s initial cell density per well and fluidity. We observe that the distribution of the conditional MSSCD depends more on the *K* than the *n*. There is a chance of overproliferation in the deterministic setup under low *n* but high *K*. The cells may die under high *n*. The cell density at maximum RPR also depends more on *K* than *n* (Fig. 4). So the cells should be cultured in the larger flask to achieve maximum proliferativeness.

**Figure 5:**
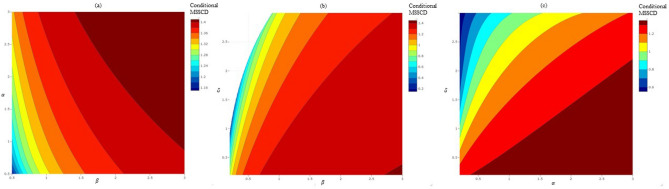
The distribution of the conditional MSSCD in parametric plane of regulators in the growth law: (**a**) dependence of the conditional MSSCD on $\alpha$ and $\beta$ parameters; (**b**) dependence of the conditional MSSCD on $\delta$ and $\beta$ parameters; (**c**) dependence of the conditional MSSCD on $\alpha$ and $\delta$ parameters.

The conditional MSSCD depends more on $\beta$ than $\alpha$ (Fig. 5a). The cells may tend to overproliferate under both high $\alpha$ and $\beta$. The conditional MSSCD does not exist for a high $\delta$ and low $\beta$ depending more on $\delta$ than $\beta$. The cells may overproliferate only under a high $\beta$ and low $\delta$ (Fig. 5b). The conditional MSSCD also depends more on $\delta$ than $\alpha$ showing mostly underproliferation of cells in the $\delta ~-\alpha$ parametric plane. Therefore, the proliferation can be controlled via regulating the interaction between the growth-inhibitory molecules and cells followed by density-regulation through contact-inhibition and cell-cell cooperation (Fig. 5c).

**Figure 6:**
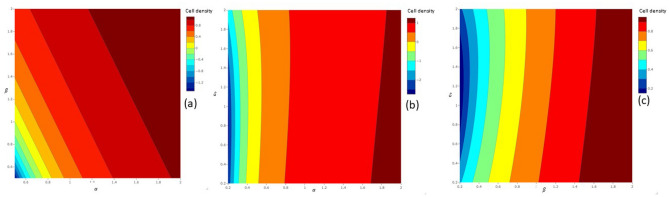
The distribution of cell density at maximum RPR in parametric plane of regulators in the growth law: (**a**) dependence on $\alpha$ and $\beta$ parameters; (**b**) dependence on $\alpha$ and $\delta$ parameters; (**c**) dependence on $\delta$ and $\beta$ parameters.

The new cell fitness measure, i.e. cell density at maximum RPR depends more on the $\alpha$ than the $\beta$ (Fig. 6a). The cells achieve maximum RPR at a great cell density under the high value of these two parameters. Figure 6b,c suggest that cell density depends only a little on the $\delta$ under high $\alpha$ and $\beta$. Under the low value of these two regulators, a high $\delta$ always reduces the cell density attaining the maximum RPR, resulting a poor cell-fitness.

### Stochastic model analysis

Our proposed stochastic model (3) can be compared with the general stratonovich stochastic differential equation $\frac{dx}{dt}=f(x)+g_{1}(x)\epsilon (t)+g_{2}(x)\Gamma (t)$. Comparing it with our proposed stochastic model we obtain $g_{1}(x)=-x^{\delta +1}$ and $g_{2}(x)=1$. Using the help of^47^, we get noise induced drift $A(x)=r_{p}x^{\alpha +1}\left( 1-\Big (\frac{x}{K}\Big )^{\beta } \right) -nx^{(\delta +1)}+D(\delta +1)x^{(2\delta +1)}-\lambda \sqrt{DQ}(\delta +1)x^{\delta }$ and noise induced diffusion coefficient $B(x)=Dx^{(2\delta +2)}-2\lambda \sqrt{DQ}x^{(\delta +1)}+Q$. The cell density at long run can be obtained from the steady state probability density function (SSPDF). The analytical expression of the SSPDF is obtained from the Fokker-Planck equation. The Fokker-Planck equation is $\frac{\partial P(x, t)}{\partial t} =- \frac{\partial \big [ A(x) P(x, t)\big ]}{\partial x}+ \frac{\partial ^{2} \big [B(x) P(x, t)\big ]}{\partial x^{2}}$, where P(x,t) is the probability density function of the cell population at the time point *t*. Solving the Fokker-Planck equation we get the SSPDF as $P_{st} (x)= \frac{N^{\prime }}{B(x)} exp \left( \int _{x} \frac{A(x^{\prime })}{B(x^{\prime })} dx^{\prime }\right)$ with the normalizing constant $N^{\prime }$. The value of $N^{\prime }$ can be obtained from $\int _{0}^{\infty } P_{st} (x)dx=1$.

This SSPDF $P_{st} (x)$ helps to understand the validity of the proposed stochastic model. Since the number of the data points is too low to fit the stochastic model to the data directly, validation of the stochastic model is challenging in this case. The dataset we used is a time series with 15 data points with three replicates only. An experiment must have many replicates to have a sample with a large sample size so that the SSPDF of cell densities obtained from theoretical findings can be validated with the real observation of cell densities at the steady state. Such datasets with many replicates are rare.

**Figure 7:**
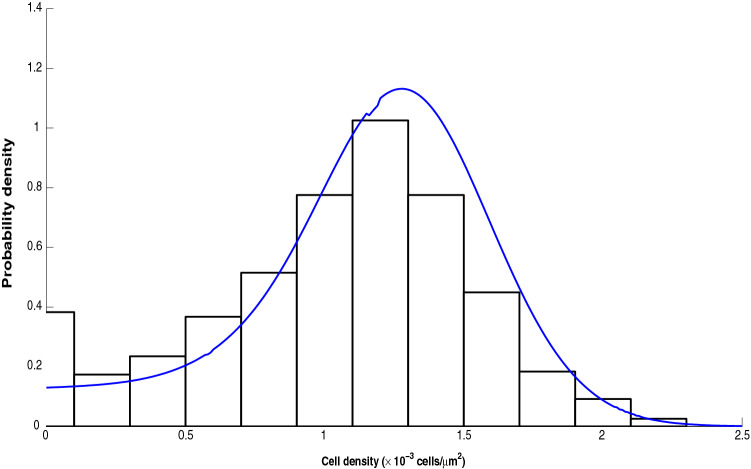
The histogram shows the distribution of cell densities at steady state under additive and multiplicative noises. The blue curve is the SSPDF. The function SSPDF and the distribution of cell densities matches to each other.

So, we generate 2000 sample paths with the help of numerical simulation based on stochastic model 3. We use the parameter values estimated from the fittings of the deterministic model to the seeding condition 1, and we consider some particular values for the two noise intensities and correlation strength ($\lambda$) to get a simulated dataset. To achieve the stationary state, we consider sufficiently large time points, and the cell densities at the final time point are used as the data set for the stationary state. We compare the frequency density of cell densities at steady-state of a simulated dataset of 2000 sample paths with the SSPDF obtained from the analytical solution. This comparison shows that the cell density distribution at the steady state matches the steady state probability density function obtained analytically (Fig. 7).

**Figure 8:**
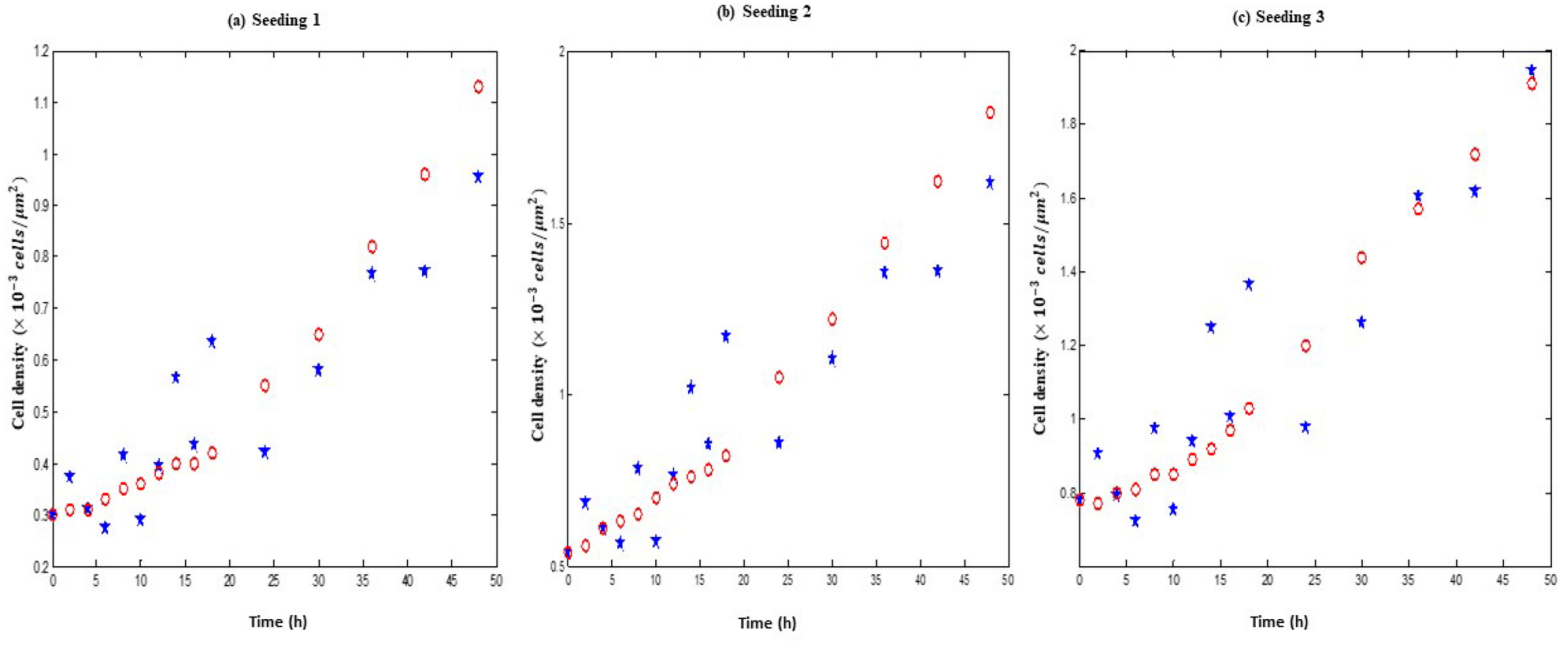
The red dots (*o*) in each sub-figures represent the experimental data of Jin et al.^1^. The blue dots ($*$) are obtained from the stochastic model (3) considering: (**a**) The seeding 1 estimated model parameters with $D= 0.002$, $Q= 0.06$ and $\lambda = 0.4$. (**b**) The seeding 2 estimated model parameters with $D= 0.01$, $Q= 0.15$ and $\lambda = 0.6$. (**c**) The seeding 3 estimated model parameters with $D= 0.002$, $Q= 0.2$ and $\lambda = 0.4$.

In addition, we illustrated the time series generated with the help of stochastic model 3 through numerical technique (Fig. 8). We have plotted the time series data thus obtained for each of the three seeding conditions and in the same figure we also plotted the observed cell densities. The red dots (*o*) represent the original/experimental dataset of Jin et al.^1^. The blue dots ($*$) represent the simulated dataset obtained from the stochastic model. This Fig. 8 clarifies our claim that the proposed stochastic model is in good agreement with the actual observation.

Figures 7 and 8 suggest that the stochastic model is valid. So the model can be further analyzed to meet the first objective. Differentiating $P_{st} (x)$, we obtain $\frac{dP_{st} (x)}{dx}=\frac{N^{\prime }}{[B(x)]^2} exp \left( \int \frac{A(x)}{B(x)}dx \right) \left( A(x)-\frac{dB(x)}{dx} \right)$ and $\frac{d^{2}P_{st} (x)}{dx^{2}}= \frac{N^{\prime }}{[B(x)]^{2}}exp \left( \int \frac{A(x)}{B(x)}dx \right) \left( \frac{dA(x)}{dx}-\frac{d^{2}B(x)}{dx^{2}} \right) +\frac{N^{\prime }}{[B(x)]^{2}} \left( A(x)-\frac{dB(x)}{dx} \right) exp \left( \int \frac{A(x)}{B(x)}dx \right) \frac{A(x)}{B(x)}-\frac{2}{[B(x)]^3}N^{\prime } exp \left( \int \frac{A(x)}{B(x)}dx \right) \left( A(x)-\frac{dB(x)}{dx} \right) \frac{dB(x)}{dx}$. At the extrema of the SSPDF, we must have $\frac{dP_{st} (x)}{dx}=0$ i.e. $\left( A(x)-\frac{dB(x)}{dx} \right) =0$.

#### **Theorem 3**

$x^{*}\approx K-K \left( \frac{nK^{\delta +1}+D(\delta +1) K^{2\delta +1}-\lambda \sqrt{DQ}(\delta +1)K^{\delta }}{\beta r K^{\alpha +1}+n(\delta +1) K^{(\delta +1)}+D(\delta +1) (2\delta +1)K^{(2\delta +1)}-\lambda \sqrt{DQ}\delta (\delta +1)K^{\delta }} \right)$ *is the conditional MSSCD due to the correlated additive and multiplicative noises under the condition* $r_{p}(\alpha +1)x^{*}{}^{\alpha }-\frac{r_{p}}{K^{\beta }}(\alpha +\beta +1)x^{*}{}^{(\alpha +\beta )} -n(\delta +1)x^{*}{}^{\delta }-D(\delta +1)(2\delta +1)x^{*}{}^{(2\delta )}+\lambda \sqrt{D\alpha }\delta (\delta +1)x^{*}{}^{(\delta -1)} < 0$ *(proof is in the* supplementary information*)*.

**Figure 9:**
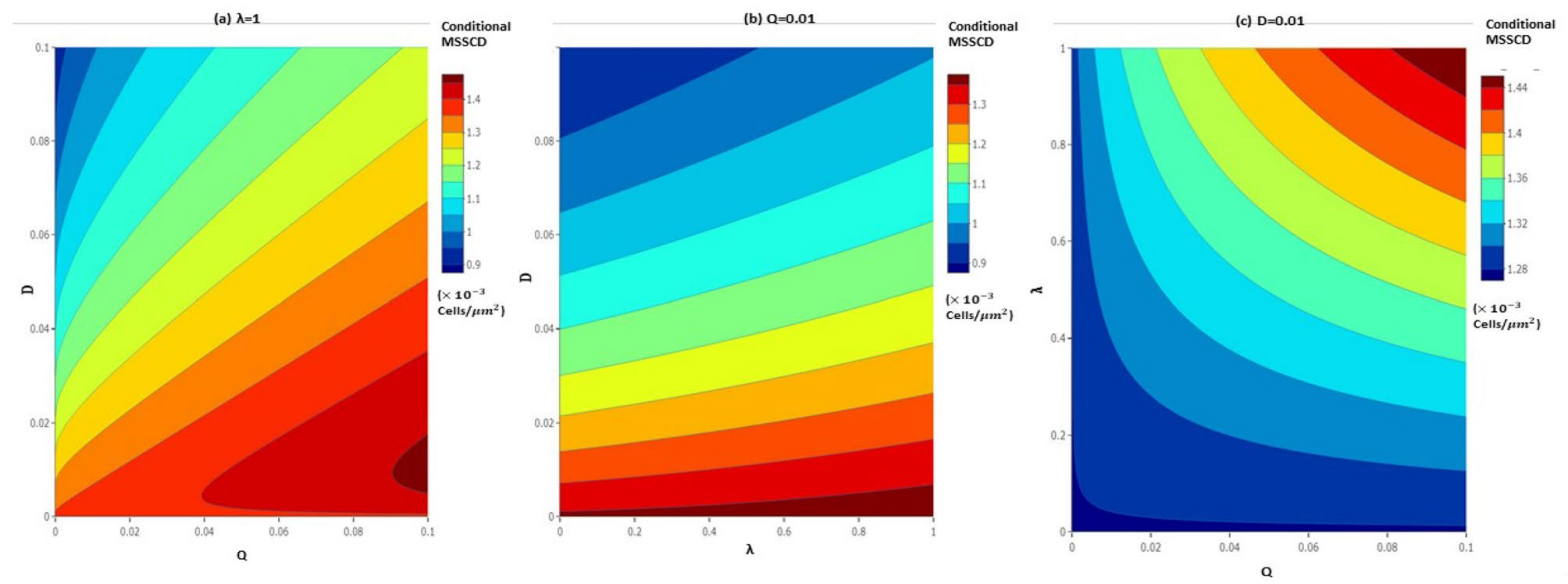
The change in the conditional MSSCD value for different noise strengths and correlation strength using the parameters estimated for seeding 1: (**a**) the conditional MSSCD values in $D-Q$ noise strength plane with highest correlation ($\lambda =1$); (**b**) the conditional MSSCD values in $D-\lambda$ noise plane with $Q=0.01$; (**c**) the conditional MSSCD values in $Q-\lambda$ noise plane with $D=0.01$.

Figure 9 visualizes the effect of noise strength and correlation strength on the conditional MSSCD. The conditional MSSCD increases with the additive noise strength (Q) and decreases with the multiplicative noise strength (D) when the other model parameters are fixed (Fig. 9a). There is a high chance of overproliferation for a low D and a high Q (Fig. 9a). Again, there is a high chance of extinction for the low Q and high D. The conditional MSSCD depends more on D than $\lambda$ (Fig. 9b), and more on $\lambda$ than Q (Fig. 9c). The conditional MSSCD increases with $\lambda$ and Q; there is a high chance of overproliferation for high $\lambda$ and Q. The extinction risk of cells from the culture increases with low $\lambda$ and Q.

**Figure 10:**
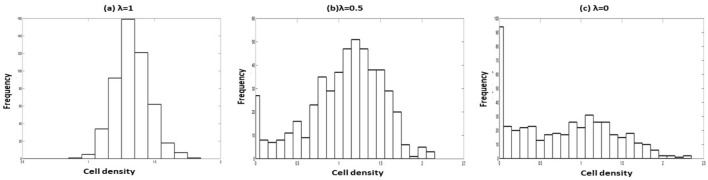
Distribution of cell density for $r_{p}=0.13$, $K=1.43$, $n=0.0095$, $\alpha =1.15$, $\beta =0.99$, $\delta =0.2$, $D=0.01$, $Q=0.01$, and variable correlation between additive and multiplicative noises: (**a**) $\lambda =1$, (**b**) $\lambda =0.5$ and (**c**) $\lambda =0$.

Due to the difficulty and complicated expression of the analytical expression of the SSPDF, we use numerical simulation to study the steady-state behavior in the long run under correlated noises. We draw a histogram of the cell densities based on 500 normal sample paths at the final time points. We use seeding 1 fitting estimates as the initial parameter values for this simulation. The cell population is stable and steady at either 0 cell density or at the conditional MSSCD. The distribution is symmetric around the conditional MSSCD for $\lambda =1$ (Fig. 10a). There is a loss in the symmetry for the decreasing $\lambda$. For $\lambda =0.5$, there is a mode at the zero states with another mode at conditional MSSCD (Fig. 10b). The histogram shows a bi-modality for low values of $\lambda$. The mode at the zero state is highest for $\lambda =0$ (Fig. 10c). Therefore, the extinction chance increases for zero noise correlation between the additive and the multiplicative noises.

**Figure 11:**
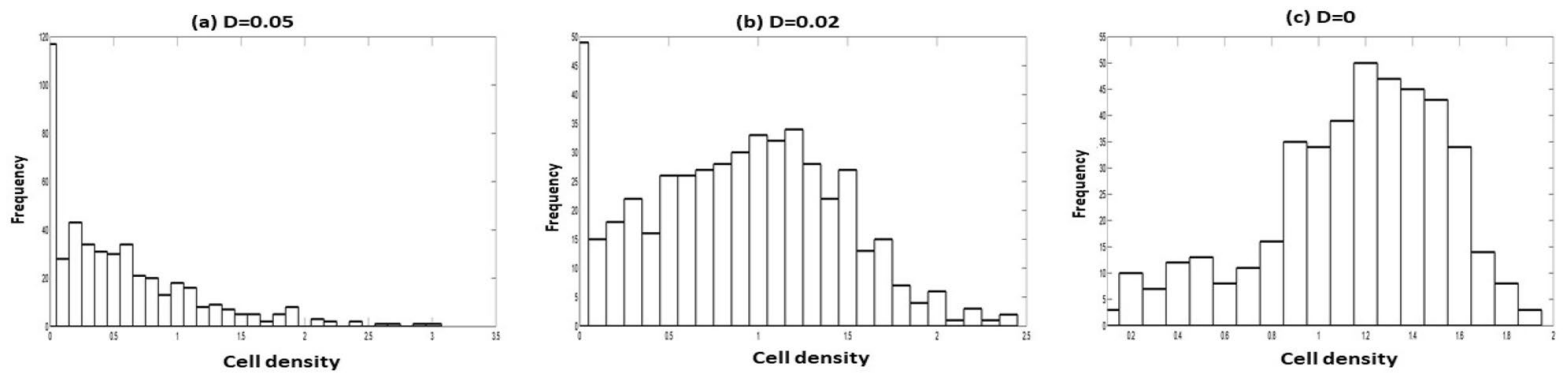
Distribution of cell density for $r_{p}=0.13$, $K=1.43$, $n=0.0095$, $\alpha =1.15$, $\beta =0.99$, $\delta =0.2$, $\lambda =0.5$, $Q=0.01$, and variable strength of multiplicative noise: (**a**) $D=0.05$, (**b**) $D=0.02$ and (**c**) $D=0$.

**Figure 12:**
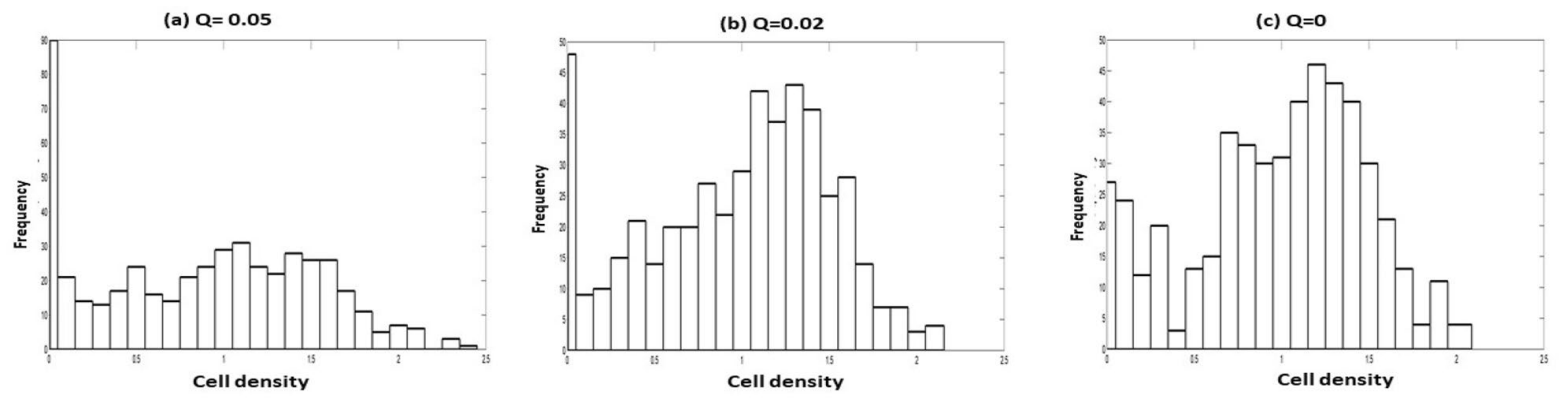
Distribution of cell density for $r_{p}=0.13$, $K=1.43$, $n=0.0095$, $\alpha =1.15$, $\beta =0.99$, $\delta =0.2$, $\lambda =0.5$, $D=0.01$, and variable correlation between multiplicative noise: (**a**) $Q=0.05$, (**b**) $Q=0.02$ and (**c**) $Q=0$.

The sustainability of the cell population depends on the strength of the two noises, like the correlation strength between them. For the zero strength multiplicative noise, the population has the mode at around the conditional MSSCD value (Fig. 11). Therefore, the population sustains in this case and tries to stabilize at the conditional MSSCD value. For $D=0.02$, there is a bimodality, where the highest mode is at the zero cell density. For $D=0.05$, we observe only one mode at $x=0$. Therefore, with the increasing values of the multiplicative noise strengths (D), the chance of extinction increases for $\lambda =0.5$, $Q=0.01$, and other constant model parameters for the seeding condition 1. Similar things happen for increasing Q values considering $D=0.01$, $\lambda =0.5$, and other constant model parameters (Fig. 12).

#### *Remark 5*

We have previously discussed the scenario for $\alpha =\delta$ for deterministic case in Remark 4. It is important to understand the scenario under stochastic case too. For $\alpha =\delta$ the proposed stochastic model 3 becomes $\frac{dx(t)}{dt}=r_{p}x(t)^{(\alpha +1)}\left( 1-\big (\frac{x(t)}{K}\big )^{\beta }\right) - nx(t)^{(\alpha +1)}-x(t)^{(\alpha +1)} \epsilon (t)+ \Gamma (t)$. For this stochastic model $g_{1}(x)=-x^{\alpha +1}$ and $g_{2}(x)=1$. We get, $A(x)=r_{p}x^{\alpha +1}\left( 1-\Big (\frac{x}{K}\Big )^{\beta } \right) -nx^{(\alpha +1)}+D(\alpha +1)x^{(2\alpha +1)}-\lambda \sqrt{DQ}(\alpha +1)x^{\alpha }$ and $B(x)=Dx^{(2\alpha +2)}-2\lambda \sqrt{DQ}x^{(\alpha +1)}+Q$. The extrema of the SPDF $\big (x(t)=x^{*}\big )$ must satisfy the growth equation $r_{p}{x^{*}}^{\alpha +1}-\frac{r_{p}}{K^{\beta }}(x^{*})^{\alpha +\beta +1}-n(x^{*})^{\alpha +1}-D(\alpha +1)(x^{*})^{2\alpha +1}+\lambda \sqrt{D~Q}(\alpha +1)(x^{*})^{\alpha }=0$.

Therefore, for $\alpha =\delta$ the conditional MSSCD is $x^{*}= K-K\frac{nK^{(\alpha +1)}+D(\alpha +1)K^{(2\alpha +1)}-\lambda \sqrt{DQ}(\alpha +1)K^{\alpha }}{\beta r_{p}K^{(\alpha +1)}+nK^{(\alpha +1)}(\alpha +1)+D(\alpha +1)(2\alpha +1)K^{(2\alpha +1)}-\alpha \lambda \sqrt{DQ}(\alpha +1)K^{\alpha }}$ under the condition $(r_{p}-n)(\alpha +1)(x^{*})^{\alpha }-\frac{r_{p}}{K^{\beta }}(\alpha +\beta +1)(x^{*})^{(\alpha + \beta )}-(\alpha +1)(2\alpha +1)D(x^{*})^{2\alpha }+\lambda \sqrt{DQ}(\alpha +1)\alpha (x^{*})^{(\alpha -1)}<0$.

#### *Remark 6*

It is meaningful to understand the mathematical difficulties involved with colored noise scenario. To observe the effect analytically, we must know the value of $f^{\prime }(x)$ at the conditional MSSCD $x(t)=K -K\left( \frac{\Big (\beta r_{p}K^{\alpha }+n \delta K^{\delta }\Big )-\sqrt{\Big (\beta r_{p}K^{\alpha }+n \delta K^{\delta }\Big )^{2}-2 \left( 2 \alpha \beta r_{p}K^{\alpha } +\beta (\beta -1)r_{p}K^{\alpha }+\delta (\delta -1)nK^{\delta } \right) nK^{\delta }}}{\left( 2 \alpha \beta r_{p}K^{\alpha } +\beta (\beta -1)r_{p}K^{\alpha }+\delta (\delta -1)nK^{\delta } \right) }\right)$ to find the functional form of the noise induced drift *A*(*x*) and the noise induced diffusion coefficient *B*(*x*) of the Fokker-Planck equation as depicted in the stochastic model analysis section. After finding these functional forms, one can similarly observe the effect of the Gaussian colored noise like the white noise. However, for the proposed deterministic model (2), observation of the colored noise is quite difficult. Therefore, for simplicity, we discuss the effect of the Gaussian white noise on cell proliferation and avoid the effect of the correlation times.

## Discussion and conclusion

We have formed a model with density-dependent IPR, regulation of environmental resistance (e.g., contact inhibition), and a mortality term similar to the harvesting but with regulatory parameter based on the concept of intercellular-interaction-induced cell proliferation and growth inhibitory molecules in the culture. This generalized extended logistic model can successfully portray the Allee effect, cooperation, and additional negative feedback for cell population. To the best of our knowledge, we are the first to introduce correlated additive and multiplicative noises in the single-dimensional generalized model for the cell proliferation. Such a correlated noise structure in the stochastic framework helps to understand the proliferating mechanism of cells in the culture where the heterogeneity in growth-inhibiting molecules may originate due to environmental fluctuations. The incorporation of regulatory parameters enables the model to be more explanatory for any cell proliferation dataset with the underlying inter-cellular cooperation, environmental resistance through contact inhibition, and the interaction between cell and growth-inhibitory molecules; it also detects the conditional MSSCD under a given environment.

In our new model, conditional MSSCD actually replaces the carrying capacity as the determinant of proliferation statuses: regular, under, and overproliferation. The theoretical exploration of the model provides a new measure of fitness. Fisher^46^ showed how the RGR (RPR for proliferating cells) could measure a population fitness. Combining the Fishers’ RGR concept and the cell proliferativeness, our proposed measure states that the fitness of proliferating cells is the density at maximum RPR. So this new fitness is no more the cell population’s inherent property only. It depends on the interaction rates of cells in between themselves or with environmental resources, growth-inhibiting factors, and inherent constant proliferation rate through the parameters. Observation of cells often showing different proliferativeness in two different setups can support this new measure of fitness.

The evaluation of our model through the scratch assay data reassures the flexibility and superiority of our proposed model over the logistic one to portray cell proliferation. The comparison of the model with previous fits to the dataset reveals that the estimated parameters are more realistic for the newly proposed model than logistic one; especially the carrying capacity is closer to the last observed data point for our model than the one used by Jin et al.^1^. The simulations using our estimated parameters explain how overproliferation, underproliferation, and regular proliferation may occur with varying parameters. Although, controlling our model’s constant inherent proliferation rate for a cell population is impossible, we find that manipulating the conditional MSSCD is plausible through the culture media amount, composition, flask size, growth-inhibiting molecule, and most importantly, the cellular-interaction rates. According to our findings, the regulator of growth-inhibitor uptake and associated negative feedback is the first target to get desired conditional MSSCD. Other interacting molecules that bind with inhibitors can be introduced for this purpose.

Another way can be to culture cells in a larger flask with a continuous flow of fresh new media. This technique may reduce the interaction time between cells and growth-inhibiting molecules, resulting in a greater value of $\delta$ followed by a greater conditional MSSCD. The distribution of the conditional MSSCD on the parametric plane changes its Kurtosis and direction after moderate cellular-cooperation, environmental resistance, and negative feedback regulation. A reason for such an observation may be hidden in cells’ changing signaling procedures affected by the environmental interaction. More research is necessary for the field of epigenetic signaling to justify the observation from simulation thoroughly. Similar to the conditional MSSCD, the simulation also detects the cell density at maximum RPR, i.e., the new fitness measure has a changing Kurtosis in the $\alpha$–$\delta$ and $\beta$–$\delta$ parametric planes. The high Kurtosis near the low value of $\alpha$ and $\beta$ suggests that the regulation of interaction growth-inhibiting molecules and cells can only affect cell fitness if the interactions between cells are low. An explanation of this observation is that the cells share the proliferative signal upon interacting between them to nullify the effect of growth-inhibiting molecules.

Introduced noises do not alter the effect of new parameters on the conditional MSSCD. In addition, the stochastic setup of the model further examines the trend in cell density distribution pattern and the conditional MSSCD for different strengths of multiplicative and additive noises and their correlation strength. Since experiments are always prone to error, the stochastic setup portrays the final and true nature of generalized growth law for cell proliferation. The finding from the stochastic modeling provides a fair idea about the chance to achieve the desired conditional MSSCD. Sacrificing in the costs of scratch assay always introduces multiplicative and additive noises in the negative feedback rate and APR, respectively^48–50^. The additive noise or the environmental fluctuation possibly causes cells to form overproliferating patches in parts of the culture plate, where they can proliferate more and eliminates from parts where the environment is adverse for proliferation. As a result, the overproliferating patch numbers are high under low multiplicative noise strength implying that the total culture is likely to experience overall overproliferation. The multiplicative noise, correlated to the additive noise, concentrates the growth-inhibiting molecules in parts of the culture flask, where the cells are decaying or underproliferating. The overproliferating patches may have diluted growth-inhibiting molecules due to the correlation between the two noises. For example, there can be heterogeneity in streptomycin and penicillin throughout the culture associated with uneven temperature in the culture flask. Thus, a high correlation strength between the noises plays a vital role in regulating conditional MSSCD to an amount near *K* or carrying capacity.

The regulatory parameters in the deterministic and stochastic models are crucial to controlling cell proliferation in empirical research. For example, cooperation or cell-cell interaction increases cell density. The plating of cells at first can be distant to reduce this interaction. Two distantly placed cells will need more time to interact and signal each other. Therefore, the distance between two cells during empirical research controls the cell-cell interaction rate parameter. The crowding regulator of the cell is also governed by the distance between two cells during plating. If the cell density is low during plating, the crowding effect will be automatically low for cells. Otherwise, regular wash with fresh media may help empirical scientists to avoid the crowding pressure. For empirical scientists, the regulation of growth-inhibiting molecules and cells may be difficult to control. This regulation depends on the cell’s property to intake the growth-inhibiting molecules and its ability to respond. So this regulation varies with growth inhibitor types and cell lines. An empirical scientist may determine the regulation rate of growth inhibition by changing the inhibitor types mainly.

An empirical researcher may aim to reduce the randomness in the scratch assay. There are several challenges in reducing this randomness in the assay. The empirical researcher needs good control of the cell culture environment, a good quality homogeneous media, and homogeneous plating of cells to avoid errors. Note that improving these criteria to reduce randomness may increase the cost of the experiment. Controlling the correlation strength ($\lambda$) between two noises is not possible manually. However, the intensities of both the additive and multiplicative noises are possible to be reduced by experimental scientists. The multiplicative noise can be reduced by making the media and cell plating more homogeneous. The growth-inhibitor molecules should be immensely vortexed in order to prevent multiplicative noise (D) due to heterogeneous growth inhibitor molecules. The additive noise occurs due to a random environment. Therefore, an experimental scientist must use better equipment to control the environment of cell culture. Otherwise, scientists can regularly monitor the environment to avoid any fluctuations in it. Also, the experimenter may perform more replicates for each experiment. We advise taking more data points after the cells reach the steady state in order to confirm that the cells have reached the steady state with the least effect of noises.

Our model, through its Theorem 3, can determine the expected conditional MSSCD in cost-effective cultures. This model serves as a tool for experimenter biologists to predict the health of a cell population and the chance of overproliferation/underproliferation. As per our objective, we have successfully determined the cell density attaining the maximum fitness and predicted the trend in conditional MSSCD for deterministic and stochastic setups using a real dataset.

## Supplementary Information

Supplementary Information.

## Data Availability

The datasets analysed during the current study are available in the Tables 4, 5, and 6 of the supplementary material of published work^1^. The link to the data is https://static-content.springer.com/esm/art%3A10.1007%2Fs11538-017-0267-4/MediaObjects/11538_2017_267_MOESM1_ESM.pdf. The softwares used in this study are R (version 4.1.3) and MATLAB (version- R2012b).

## Code availability

The codes will be available on requests.
