# Supplementary material for: A noble extended stochastic logistic model for cell proliferation with density-dependent parameters
Source: Sci Rep. 2022 May 30;12:8998. doi: 10.1038/s41598-022-12719-y (PMC9151920; doi:10.1038/s41598-022-12719-y)
Supplement: Supplementary file 1 — Supplementary Information. [file 41598_2022_12719_MOESM1_ESM.pdf]

Supplementary Information

Proof of Theorem 1

Proof. Power series approximation is a well known technique to find a root near $x(t)=K$, given $|\frac{x(t)-K}{K}|<1$ is satisfied. This condition is obvious for any sigmoidal growth curve model with a carrying capacity (K). So we expand each allometric power of $x(t)$ of the equation $\left(r_p x(t)^{(\alpha)}\left(1-(\frac{x(t)}{K})^{\beta}\right)-nx(t)^{(\delta)}\right)=0$ up to second degree terms and ignore the highers. Finally, we get a quadratic equation

$$\left(\frac{x(t)-K}{K}\right)^2\times\left(\frac{\alpha\beta}{2}r_pK^{\alpha}+\frac{\beta(\alpha+\beta-1)}{2}r_pK^{\alpha}+\frac{\delta(\delta-1)}{2}nK^{\delta}\right)+\left(\frac{x(t)-K}{K}\right)\times\left(\beta r_pK^{\alpha}+n\delta K^{\delta}\right)+nK^{\delta}=0.$$

Solving this quadratic equation, we derive two roots, suggesting two cell densities. Among them,

$$x^*=K-K\left(\frac{\left(\beta r_pK^{\alpha}+n\delta K^{\delta}\right)-\sqrt{\left(\beta r_pK^{\alpha}+n\delta K^{\delta}\right)^2-2\left(2\alpha\beta r_pK^{\alpha}+\beta(\beta-1)r_pK^{\alpha}+\delta(\delta-1)nK^{\delta}\right)nK^{\delta}}}{\left(2\alpha\beta r_pK^{\alpha}+\beta(\beta-1)r_pK^{\alpha}+\delta(\delta-1)nK^{\delta}\right)}\right)$$

is the maximum cell density around the carrying capacity and

$$x^*=K-K\left(\frac{\left(\beta r_pK^{\alpha}+n\delta K^{\delta}\right)+\sqrt{\left(\beta r_pK^{\alpha}+n\delta K^{\delta}\right)^2-2\left(2\alpha\beta r_pK^{\alpha}+\beta(\beta-1)r_pK^{\alpha}+\delta(\delta-1)nK^{\delta}\right)nK^{\delta}}}{\left(2\alpha\beta r_pK^{\alpha}+\beta(\beta-1)r_pK^{\alpha}+\delta(\delta-1)nK^{\delta}\right)}\right)$$

is the minimum cell density. The minimum one is the approximated conditional threshold population cell density and the maximum one is the conditional MSSCD. These two points exists if $\left(\beta r_pK^{\alpha}+n\delta K^{\delta}\right)^2-2\left(2\alpha\beta r_pK^{\alpha}+\beta(\beta-1)r_pK^{\alpha}+\delta(\delta-1)nK^{\delta}\right)nK^{\delta}\geq 0.$ □

We validate our proposed deterministic model with three sets of scratch assay cell proliferation data sets. The estimated model parameters satisfy $\left(\beta r_pK^{\alpha}+n\delta K^{\delta}\right)^2-2\left(2\alpha\beta r_pK^{\alpha}+\beta(\beta-1)r_pK^{\alpha}+\delta(\delta-1)nK^{\delta}\right)nK^{\delta}\geq 0$ for all of the three seeding conditions (Table 1 in the supplementary material). This condition is for the existence of the critical threshold density and the conditional MSSCD.

**Seeding condition**	$\left(\beta r_pK^{\alpha}+n\delta K^{\delta}\right)^2-2\left(2\alpha\beta r_pK^{\alpha}+\beta(\beta-1)r_pK^{\alpha}+\delta(\delta-1)nK^{\delta}\right)nK^{\delta}$
1	0.0295
2	0.0318
3	0.0459

Table 1. The estimated model parameters for all of the seeding conditions satisfied the existence of the conditional threshold and the conditional MSSCD.

Effective potential function estimation

$$f(x)\left(=r_px(t)^{(\alpha+1)}\left(1-(\frac{x(t)}{K})^{\beta}\right)-nx(t)^{(\delta+1)}\right)$$

is the deterministic force controlling this cell proliferation dynamics. Let $U(x)$ be the effective potential for this deterministic force with the following analytical form-

$$\begin{aligned}U(x)&=-\int f(x)dx\\&=-\left(\frac{r_p}{\alpha+2}x^{\alpha+2}-\frac{r_p}{K^{\beta}(\alpha+\beta+2)}x^{\alpha+\beta+2}-\frac{nx^{\delta+2}}{\delta+2}\right).\end{aligned}$$

The potential function has a deeper well at the conditional MSSCD cell count and flatter well at around the zero cell count (figure 1). At the red dotted line of the figure 1,

$$x(t)=K-K\left(\frac{\left(\beta r_pK^{\alpha}+n\delta K^{\delta}\right)-\sqrt{\left(\beta r_pK^{\alpha}+n\delta K^{\delta}\right)^2-2\left(2\alpha\beta r_pK^{\alpha}+\beta(\beta-1)r_pK^{\alpha}+\delta(\delta-1)nK^{\delta}\right)nK^{\delta}}}{\left(2\alpha\beta r_pK^{\alpha}+\beta(\beta-1)r_pK^{\alpha}+\delta(\delta-1)nK^{\delta}\right)}\right).$$

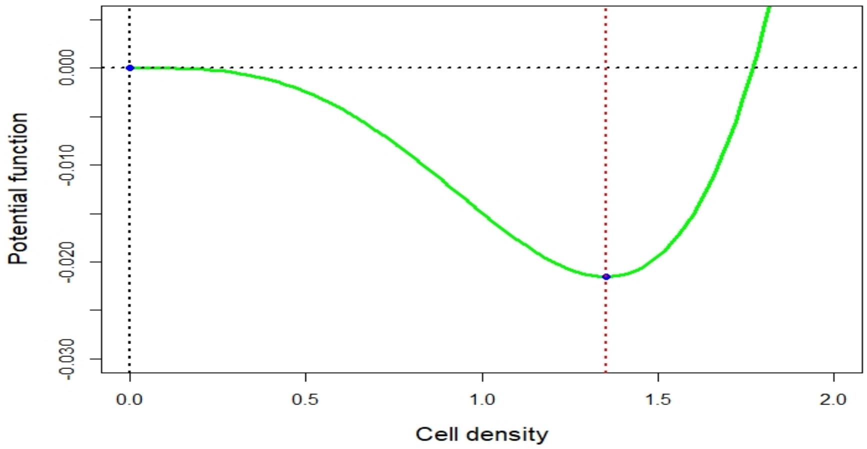

Figure 1. The potential function has the minima at $x(t) = 1.351$. The minima occurs at the MSSCD count.

This validates the populations sustainability at this conditional MSSCD cell count for the seeding condition 1.

Proof of Theorem 2

Proof. The RPR value of the proposed model is $R(t) = \frac{1}{x(t)}\frac{dx(t)}{dt} = r_p x(t)^{\alpha}\left(1-(\frac{x(t)}{K})^{\beta}\right) - nx(t)^{\delta}$. Therefore, $\frac{dR(t)}{dx(t)} = r_p\alpha x(t)^{(\alpha-1)} - \frac{r_p}{K^{\beta}}(\alpha+\beta)x(t)^{(\alpha+\beta-1)} - n\delta x(t)^{\delta-1}$ and $\frac{d^2R(t)}{dx(t)^2} = r_p\alpha(\alpha-1)x(t)^{(\alpha-2)} - \frac{r_p}{K^{\beta}}(\alpha+\beta)(\alpha+\beta-1)x(t)^{(\alpha+\beta-2)} - n\delta(\delta-1)x(t)^{\delta-2}$. At the extreme RPR values, $\frac{dR(t)}{dx(t)} = 0$. The power series approximation for each of the allometric powers of $x(t)$ around the carrying capacity K in the equation $r_p\alpha x(t)^{(\alpha-1)} - \frac{r_p}{K^{\beta}}(\alpha+\beta)x(t)^{(\alpha+\beta-1)} - n\delta x(t)^{\delta-1} = 0$ up to first degree and ignoring the higher degree terms suggests $x(t) = K - K\left(\frac{r_p\beta K^{(\alpha-1)}+n\delta K^{(\delta-1)}}{2r_p\alpha\beta K^{(\alpha-1)}+r_p\beta(\beta-1)K^{(\alpha-1)}+n\delta(\delta-1)K^{(\delta-1)}}\right)$. Therefore, the extreme RPR value will be maximum at the density $x^* = K - K\left(\frac{r_p\beta K^{(\alpha-1)}+n\delta K^{(\delta-1)}}{2r_p\alpha\beta K^{(\alpha-1)}+r_p\beta(\beta-1)K^{(\alpha-1)}+n\delta(\delta-1)K^{(\delta-1)}}\right)$ if it satisfies the condition $\frac{d^2R(t)}{dx(t)^2} < 0$ at the point i.e.
$r_p\alpha(\alpha-1)x^{*(\alpha-2)} - \frac{r_p}{K^{\beta}}(\alpha+\beta)(\alpha+\beta-1)x^{*(\alpha+\beta-2)} - n\delta(\delta-1)x^{*\delta-2} < 0$. In that case, the RPR profile must be concave downward with maximum RPR value at $x(t) = x^*$. □

Stability analysis of the proposed deterministic model for $\alpha = \delta$

At the equilibrium point,the proposed model 2 must satisfy the growth equation $r_p x(t)^{(\alpha+1)}\left(1-(\frac{x(t)}{K})^{\beta}\right) - nx(t)^{(\alpha+1)} = 0$ i.e. $x(t)^{\alpha+1}\times\left[r_p\left(1-(\frac{x(t)}{K})^{\beta}\right)-n\right] = 0$ for the case $\alpha = \delta$. Therefore $x(t) = 0$ is one equilibrium point and the other equilibrium point must satify $r_p\left(1-(\frac{x(t)}{K})^{\beta}\right)-n$. Solving this equation we get $x(t) = K(1-\frac{n}{r_p})^{\frac{1}{\beta}}$. For $\alpha = \delta$, at $x(t) = K(1-\frac{n}{r_p})^{\frac{1}{\beta}}$ we get $\frac{dx(t)}{dt} = 0$ and $\frac{d^2x(t)}{dt^2} = (r_p-n)(\alpha+1)K^{\alpha}(1-\frac{n}{r_p})^{\frac{\alpha}{\beta}} - r_p(\alpha+\beta+1)K^{\alpha}(1-\frac{n}{r_p})^{\frac{\alpha}{\beta}+1}$. In this case $\frac{d^2x(t)}{dt^2} < 0$ at $x(t) = K(1-\frac{n}{r_p})^{\frac{1}{\beta}}$ for $r_p > n$. Therefore, $x(t) = K(1-\frac{n}{r_p})^{\frac{1}{\beta}}$ is a stable equilibrium point and $x(t) = 0$ is an unstable equilibrium point for $r_p > n$.

For $\alpha = \delta$, the RPR value of the proposed model is $R(t) = \frac{1}{x(t)}\frac{dx(t)}{dt} = r_p x(t)^{\alpha}\left(1-(\frac{x(t)}{K})^{\beta}\right) - nx(t)^{\alpha}$. Therefore, $\frac{dR(t)}{dx(t)} = (r_p-n)\alpha x(t)^{(\alpha-1)} - \frac{r_p}{K^{\beta}}(\alpha+\beta)x(t)^{(\alpha+\beta-1)}$ and $\frac{d^2R(t)}{dx(t)^2} = (r_p-n)\alpha(\alpha-1)x(t)^{(\alpha-2)} - \frac{r_p}{K^{\beta}}(\alpha+\beta)(\alpha+\beta-1)x(t)^{(\alpha+\beta-2)}$. Solving $\frac{dR(t)}{dx(t)} = 0$ we get, $x(t) = K(\frac{(r_p-n)\alpha}{r_p(\alpha+\beta)})^{\frac{1}{\beta}}$. The RPR profile will be concabe downward with the maximum RPR at $x(t) = K(\frac{(r_p-n)\alpha}{r_p(\alpha+\beta)})^{\frac{1}{\beta}}$ for $r_p > n$.

Proof of Theorem 3

Proof. At the conditional MSSCD of the proposed stochastic model (3), $\left(A(x)-\frac{dB(x)}{dx}\right)=0$ and $\frac{d^2P_{st}(x)}{dx^2}<0$. Therefore, $P_{st}(x)$ is maximum at the density x^* satisfying the equation $r_p x^{*(\alpha+1)}-\frac{r_p}{K\beta}x^{*(\alpha+\beta+1)}-nx^{*(\delta+1)}-D(\delta+1)x^{*(2\delta+1)}+\lambda\sqrt{DQ}(\delta+1)x^{*(\delta+1)}=0$. Using the power series approximation upto first degree around K we obtain the solution as $x^*=K-K\left(\frac{nk^{\delta+1}+D(\delta+1)K^{2\delta+1}-\lambda\sqrt{DQ}(\delta+1)K^{\delta}}{\beta rK^{\alpha+1}+n(\delta+1)K^{(\delta+1)}+D(\delta+1)(2\delta+1)K^{(2\delta+1)}-\lambda\sqrt{DQ}\delta(\delta+1)K^{\delta}}\right)$. This x^* is the MSSCD under the condition $\frac{dA(x)}{dx}-\frac{d^2B(x)}{dx^2}<0$ i.e. $r_p(\alpha+1)x^{*\alpha}-\frac{r_p}{K\beta}(\alpha+\beta+1)x^{*(\alpha+\beta)}-n(\delta+1)x^{*\delta}-D(\delta+1)(2\delta+1)x^{*(2\delta)}+\lambda\sqrt{DQ}\delta(\delta+1)x^{*(\delta-1)}<0$. □

For the numerical simulation to study the steady state behavior in the long run, we consider different strength of the noises. For each combination of the strength of the noises the above condition ($\frac{dA(x)}{dx}-\frac{d^2B(x)}{dx^2}<0$ i.e. $r_p(\alpha+1)x^{*\alpha}-\frac{r_p}{K\beta}(\alpha+\beta+1)x^{*(\alpha+\beta)}-n(\delta+1)x^{*\delta}-D(\delta+1)(2\delta+1)x^{*(2\delta)}+\lambda\sqrt{DQ}\delta(\delta+1)x^{*(\delta-1)}<0$) is satisfied (Table 2 in the supplementary material).

**Seeding condition**	D	λ	Q	$r_p(\alpha+1)(x^*)^{\alpha}-\frac{r_p}{K\beta}(\alpha+\beta+1)(x^*)^{(\alpha+\beta)}-n(\delta+1)(x^*)^{\delta}$ $-D(\delta+1)(2\delta+1)(x^*)^{(2\delta)}+\lambda\sqrt{D\alpha}\delta(\delta+1)(x^*)^{(\delta-1)}$
1	0.01	1	0.01	−0.1705
1	0.01	0.5	0.01	−0.1569
1	0.01	0	0.01	−0.1440
1	0.05	0.5	0.01	−0.1362
1	0.02	0.5	0.01	−0.1455
1	0	0.5	0.01	−0.1455
1	0.01	0.5	0.05	−0.1738
1	0.01	0.5	0.02	−0.1625
1	0.01	0.5	0	−0.1440

Table 2. For all of the noise strengths the condition for the existence of the conditional MSSCD is satisfied.
